# Evaluation of laboratory values affecting mortality of end-stage renal disease patients: a competing risks approach

**DOI:** 10.1186/s12882-023-03234-x

**Published:** 2023-07-18

**Authors:** Rayka Malek, Ahmadreza Baghestani, Farin Rashid-Farokhi, Shadi Shafaghi, Farzanehsadat Minoo, Foolad Eghbali, Navin Chandra, Masoud Shafaghi, Kaveh Bonyadi, Fatemeh Sadat Hosseini-Baharanchi

**Affiliations:** 1 School of Population Health sciences, King’s College London, London, UK; 2 Department of Biostatistics, Faculty of Paramedical Sciences, Shahid Beheshti University of Medical Sciences, Tehran, Iran; 3 Telemedicine Research Center, & Chronic Kidney Disease Research Center, Masih Daneshvari Hospital, Shahid Beheshti University of Medical Sciences, Tehran, Iran; 4 Lung Transplantation Research Center, National Research Institute of Tuberculosis and Lung Diseases, Shahid Beheshti University of Medical Sciences, Tehran, Iran; 5 Center of Excellence in Nephrology, Nephrology Research Center, Tehran University of Medical Sciences, Tehran, Iran; 6 Minimally Invasive Surgery Research Center, Iran University of Medical Sciences, Tehran, Iran; 7 Department of Statistics, Ramanujan School of Mathematical Sciences, Pondicherry University, Puducherry, India; 8 Strategic Planning and Executive Office Manager, International Federation of Inventors’ Associations, Geneva, Switzerland; 9 Department of Biomedical (Biomechanics), Science and Research Branch, Islamic Azad University, Tehran, Iran; 10 Department of Biostatistics, School of Public Health, Iran University of Medical Sciences, Tehran, Iran

**Keywords:** Renal insufficiency, Survival analysis, Competing risks, Kidney transplantation, Bayesian analysis

## Abstract

**Background:**

Chronic Kidney Disease (CKD) is a prevalent and life-threatening situation recognized as an emerging health issue. The present study aimed to evaluate the effect of demographic and laboratory parameters on the survival of patients with End-Stage Renal Disease (ESRD) in a hemodialysis (HD) center in Iran.

**Materials and methods:**

This study was conducted on patients receiving chronic HD in Iran Helal Pharmaceutical and Clinical Complex between 2014 and 2018. The survival time was considered as the time interval between HD initiation and death. Receiving kidney transplantation was regarded as a competing risk, and an improper form of two-parameter Weibull distribution was utilized to simultaneously model the time to both death and renal transplantation. The Bayesian approach was conducted for parameters estimation.

**Results:**

Overall, 29 (26.6%) patients expired, and 19 (17.4%) received kidney transplants. The male gender was related to poor survival, having nearly 4.6 folds higher hazard of mortality (90% HPD region: 1.36–15.49). Moreover, Serum calcium levels $\ge$9.5 mg/dL (adjusted Sub-hazard ratio (S-HR)=2.33, 90% HPD region: 1.05–5.32) and intact parathyroid hormone (iPTH) $\le$150 pg/mL (adjusted S-HR = 2.56, 90% HPD region: 1.09–6.15) were associated with an elevated hazard of mortality. The cumulative incidence function (CIF) for transplantation was greater than death in the first two years of the study. Subsequently, the CIF for death exceeded transplantation in the following two years. The 4-year cumulative incidence of death and kidney transplantation was 63.7% and 36.3%, respectively.

**Conclusion:**

Male gender, hypercalcemia, and hypoparathyroidism were associated with worse outcomes. Correcting mentioned laboratory parameters may improve patients’ survival in the HD population.

## Introduction

Chronic Kidney Disease (CKD) and its progression to End-Stage Renal Disease (ESRD) is now widely acknowledged as a substantial risk factor for death and is therefore given undeniable global public priority [1].

The burden of CKD continues to rise in the world, especially in low- and middle-income countries (LMICs). In LMICs, where developed health resources are typically scarce, the implications of CKD may be more devastating [2]. In Iran, the overall prevalence of CKD is estimated at about 15%, which is higher than the global prevalence based on a systematic review [3]. Hemodialysis (HD) is the major type of renal replacement therapy for ESRD, yet there are only a few studies conducted on the survival of these patients in developing countries [4]. The 5-year survival rate of CKD patients in the Iranian HD population was estimated at 25% [5], whereas the figure for these patients was reported 42% according to the United States Renal Data System (USRDS) [6].

The impact of variables related to CKD mineral and bone disorders (CKD-MBD) such as serum calcium, phosphorus, and intact parathyroid hormone (iPTH) levels on survival may vary by ethnic group. This is probably related to the variation in population demographics, lifestyle, and socioeconomic status [7]. Therefore, it is critical to study the survival of ESRD patients in Iran to provide better understanding of their prognosis.

HD, peritoneal dialysis (PD), and kidney transplantation are three therapeutic options in ESRD patients when estimated glomerular filtration rate (eGFR) falls below 15 mL/min/1.73m^2^ [8]. In studying survival of HD patients, receiving transplantation may alter the probability of observing death from ESRD. In medical researches, competing risks such as transplantation could be defined as an event that inhibits the observation of the event of interest, such as death, or alters its probability of occurrence [9]. Ignoring competing risks and using conventional methods such as the Kaplan–Meier (KM) method and standard Cox proportional hazards (PH) regression results in biased estimates [10]. Additionally, most competing risks methods presume competing events (such as death and transplantation) are independent of each other. This assumption is neither accurate nor realistic and concludes noninformative interpretations. The aim of current study was to investigate the impact of demographic and laboratory parameters on survival of ESRD patients in Iran through modelling competing risks using an improper form of two-parameter Weibull distribution [11]. The correlation between the competing events was also taken into account to provide more precise estimates.

## Materials and methods

### Patients

In this retrospective cohort study, all ESRD patients who received chronic HD in the Iran Helal Pharmaceutical and Clinical Complex during 2014–2018 were enrolled (no sampling method was used). The inclusion criteria were age higher than 18 years old, and routine dialysis started at least three months before the study. The exclusion criteria were having no evidence of CKD or improvement in kidney function after dialysis sessions. Demographics and laboratory results were collected from hospital medical records. HD patients were referred to the dialysis department on a regular basis as planned. Patients who did not refer were followed by telephone and in case of death or transplantation information was recorded. Patients who were unable to complete their follow-up information during the study were excluded. The survival time was calculated as the time interval between starting dialysis and the time of death. Renal transplantation was considered as a competing event.

### Measurements

At first, demographic information was gathered. After 12–14 h on an empty stomach, a blood sample was collected and centrifuged within 30–45 min of collection. The enzymatic colorimetric method with cholesterol esterase-cholesterol oxidase and glycerol phosphate oxidase were used to assess total cholesterol and triglycerides, respectively. Jaffe kinetic colorimetric analysis was used to determine serum creatinine levels. Serum uric acid, serum glutamic-oxaloacetic transaminase (SGOT), serum glutamic-pyruvic transaminase (SGPT), fasting blood sugar (FBS), alkaline phosphatase (ALP), calcium, phosphorous, iPTH, albumin, hemoglobin, ferritin, potassium, and sodium were also measured. All biochemical tests were performed using a Selectra 2 auto-analyzer with commercial kits (Pars Azmoon Inc., Tehran, Iran) (Vital Scientific, Spankeren, the Netherlands).

### Statistical analysis

Demographic and laboratory values were summarized using descriptive statistics, including mean ± standard deviation (SD) and frequency. The sub-distribution hazard rates for death and transplantation were plotted. An improper form of two-parameter Weibull distribution [11] was utilized for considering the event of interest (death) and the competing event (transplantation) simultaneously. Additionally, the correlation between the two events was taken into account assuming P(δ = 1) + P(δ = 2) = 1. For assessing PH assumption, Schoenfeld residuals test were performed, and Cox-Snell residuals plots were inspected. Missing values in independent variables were handled using multiple imputation with Expectation-Maximization (EM) algorithm. Our primary aim was to evaluate the effect of demographic and laboratory values at baseline on the mortality of ESRD patients in the presence of kidney transplantation. Bayesian analysis was conducted to estimate parameters. We assumed improper priors for predictors’ parameters and Jeffreys-type prior for the scale parameter of Weibull distribution. MCMC method and Gibs sampling algorithm were used to draw a sample from the joint posterior distribution. Univariate and multivariable analyses were performed at the 2% and 1% alpha levels, respectively, and the highest posterior density (HPD) regions were assessed. All analyses were conducted using WinBUGS and RStudio version 4.0.3.

**Fig. 1:**
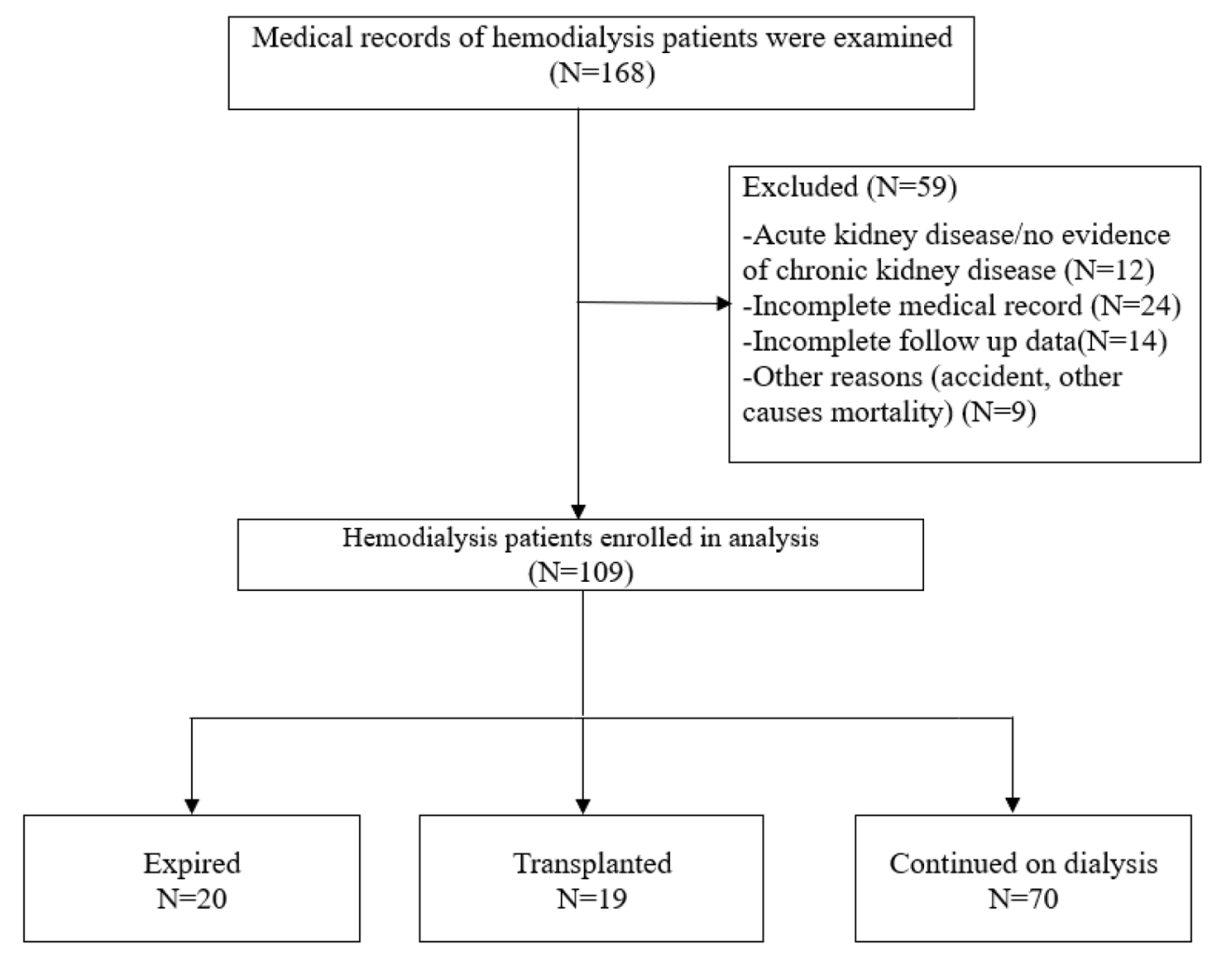
Study flow diagram of Hemodialysis patients

**Fig. 2:**
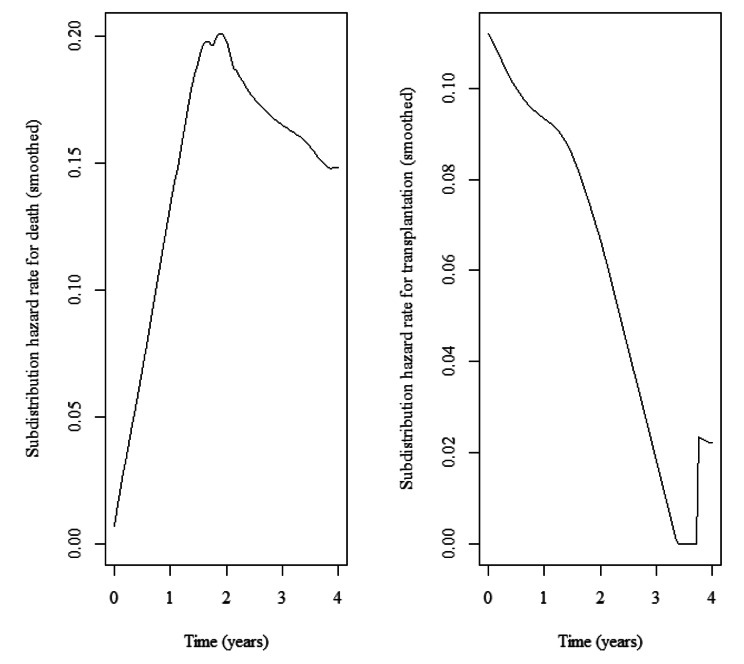
The estimate of sub-distribution hazard rates for death and transplantation

## Results

**Fig. 3:**
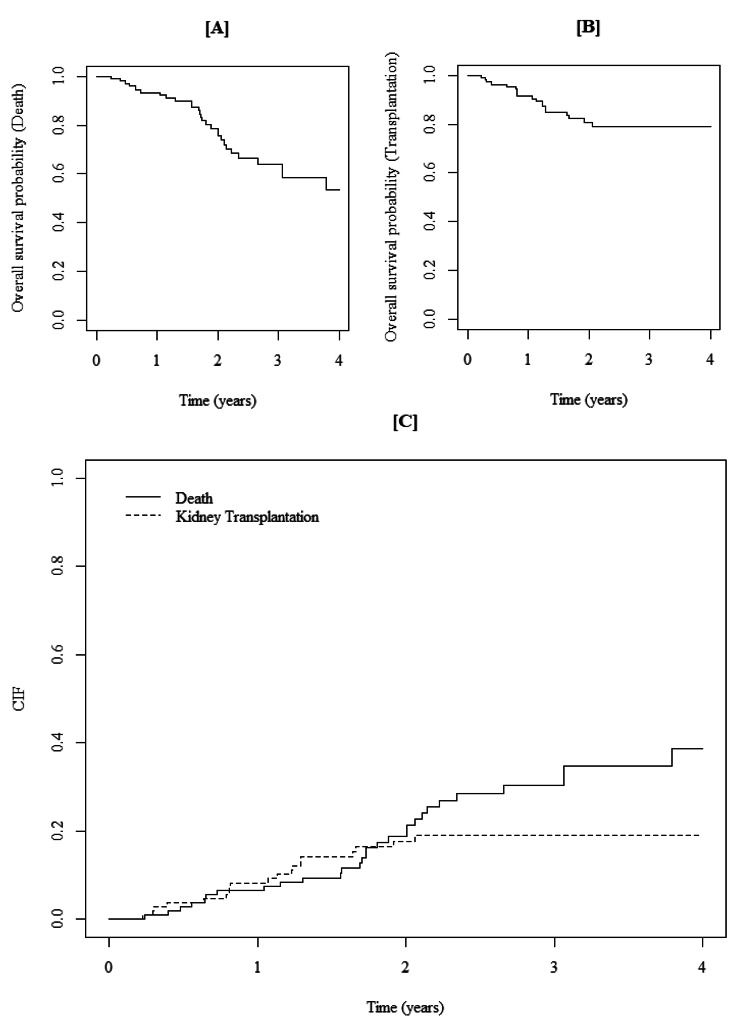
Kaplan-Meier and Cumulative Incidence function/estimate of death and transplantation

The study examined 109 HD patients (71.56% were male) with mean$\pm$SD age of 57.86$\pm$17.1. Totally, with a median (min-max) follow up time of 1.82 (0.22-4) years, 29 (26.61%) patients expired (the primary cause of interest), 19 (17.43%) patients experienced transplantation (competing risk), and others (55.96%) were censored (Fig. 1). The mean ± SD of Kt/V (an index of the removal efficiency per dialysis session) was 1.2$\pm$0.2, and had no significant difference among patients. Demographic data and laboratory values of patients are summarized in Table 1. Appropriateness of Weibull distribution was explored by plotting hazard rate for each event. The sub-distribution hazard functions for competing risks illustrated an increasing trend for death and a decreasing trend for transplantation over time (Fig. 2). KM plots are presented in Fig. 3[A] and 3[B]. As shown in Fig. 3[C], the cumulative incidence function (CIF) for transplantation was greater than death in the first two years of the study. Subsequently, the CIF for death exceeded transplantation in the following two years. The 4-year cumulative incidence of death (kidney transplant) in these patients was estimated at 63.7% (36.3%).

**Table 1:** Demographic Characteristics and Laboratory Values of ESRD Patients Undergoing Hemodialysis

Variable		Mean$\pm$SD		
		All Patients	Non-survived Patients	Patients who Underwent Transplantation
Age (years)		57.86$\pm$17.1	62.69$\pm$16.34	59.58$\pm$14.97
Cholesterol (mg/dL)		148.89$\pm$38.95	137.7$\pm$30.54	154.98$\pm$49.89
Triglycerides (mg/dL)		142.03$\pm$59	135.62$\pm$59.34	143.89$\pm$54.26
Uric Acid (mg/dL)		7.00$\pm$1.43	6.64$\pm$1.05	6.97$\pm$1.51
SGOT (U/L)		18.45$\pm$7.88	19.60$\pm$8.74	18.38$\pm$5.95
SGPT (U/L)		22.11$\pm$14.38	21.17$\pm$8.65	24.05$\pm$12.3
Ferritin (ng/mL)		411.40$\pm$362.43	432.02$\pm$292.16	345.90$\pm$293.73
Creatinine (mg/dL)		8.91$\pm$3.62	8.86$\pm$3.27	8.90$\pm$3.7
FBS (mg/dL)		118.55$\pm$64.89	129.62$\pm$90.38	108.22$\pm$45.72
Sodium (mEq/L)		142.30$\pm$2.63	142.38$\pm$2.29	141.93$\pm$2.87
N (%)				
Sex	Male	78 (71.56)	27 (93.10)	15 (78.95)
Hemoglobin (g/dL)				
	≤ 10	37 (33.94)	9 (31)	7 (36.84)
	10-12.5	49 (44.95)	11 (38)	8 (42.11)
	> 12.5	23 (21.1)	9 (31)	4 (21.05)
Potassium (mEq/L)				
	3.5-5	48 (44)	12 (41.38)	10 (52.63)
	> 5	61 (56)	17 (58.62)	9 (47.37)
ALP(U/L)				
	≤ 300	60 (55)	16 (55.17)	10 (52.63)
	> 300	49 (45)	13 (44.83)	9 (47.37)
Calcium (mg/dL)				
	≤ 8.6	19 (17.43)	6 (20.69)	3 (15.79)
	8.6–9.5	76 (69.72)	17 (58.62)	14 (73.68)
	≥ 9.5	14 (12.84)	6 (20.69)	2 (10.53)
Phosphorus(mg/dL)				
	< 5	63 (57.8)	18 (62.07)	10 (52.63)
	≥ 5	46 (42.2)	11 (37.93)	9 (47.37)
iPTH (pg/mL)				
	≤ 150	14 (12.84)	5 (17.24)	3 (15.79)
	150–600	69 (63.3)	17 (58.62)	12 (63.16)
	> 600	26 (23.85)	7 (24.14)	4 (21.05)
Albumin (g/dL)				
	< 4	27 (24.77)	9 (31.03)	3 (15.97)
	≥ 4	82 (75.23)	20 (68.97)	16 (17.43)

SD: Standard deviation; SGOT: serum glutamic-oxaloacetic transaminase; SGPT: serum glutamic-pyruvic transaminase; FBS: fasting blood sugar; ALP: Alkaline Phosphatase; iPTH: intact parathyroid hormone

Considering death as the primary event of interest (Table 2), age, being male, cholesterol, and iPTH had a significant effect on the survival of ESRD patients in the univariate analyses. An inverse relationship between phosphorous levels and HD patients’ survival was detected in the univariate model. Patients with phosphorus level $\ge$5 mg/dL had an elevated hazard of mortality compared to patients with phosphorus level $<$5 mg/dL (S-HR = 1.15 (80% HPD region: 0.69–1.82); however, it was not statistically significant.

In the multivariable analysis, assuming that the effects of all the other variables were constant, the adjusted sub-hazard ratio (S-HR) of sex was 4.64 (90% HPD region: 1.36–15.49), implying that being male had a 4.64-fold increase in the hazard of death. Moreover, both lower and higher levels of serum calcium were associated with increased mortality risk with respect to the reference group (8.6–9.5 mg/dL); however, this incremental effect was only significant in individuals with serum calcium $\ge$9.5 mg/dL (S-HR = 2.33 (90% HPD region: 1.05–5.32)).

Furthermore, patients with iPTH $\le$150 pg/mL had a 2.56-fold higher sub-hazard of mortality than individuals with iPTH between 150 and 600 pg/mL (90% HPD region: 1.09–6.15).

**Table 2:** Univariate and Multivariable Competing Risks Model for Death in ESRD Patients (n = 109)

Variables		Univariate	Multivariable	
		S-HR (80% HPD Region)		Adjusted S-HR (90% HPD Region)
Age		1.02 (1.004–1.04)*		1.02 (0.99–1.04)
Ferritin		1 (0.99–1.001)		
Creatinine		0.96 (0.92–1.01)		
Cholesterol		0.99 (0.98–0.997)*		0.99 (0.98–1.001)
Uric acid		0.89 (0.76–1.06)		
SGOT		1.02 (0.99–1.05)		
SGPT		0.98 (0.96–1.01)		
‌Sodium		0.98 (0.89–1.07)		
‌FBS		1 (0.99–1.005)		
‌Triglycerides		0.997 (0.99–1.002)		
Sex	Male	5.84 (1.97–15.26)*		4.64 (1.36–15.49)**
Hemoglobin				
	$\le$10	1.02 (0.59–1.84)		
	10-12.5 > 12.5	1 1.58 (0.89–2.87)		
Potassium				
	3.5–5.5	1		
	> 5	1.03 (0.63–1.66)		
ALP				
	$\le$300	1		
	> 300	0.93 (0.59–1.51)		
Calcium				
	$\le$8.6	1.23 (0.68–2.31)		1.26 (0.54–3.02)
	8.6–9.5 $\ge$9.5	1 1.64 (0.91–3.14)		-- 2.33 (1.05–5.32)**
Phosphorus				
	$<$5	1		
	$\ge$5	1.15 (0.69–1.82)		
iPTH				
	$\le$150	1.97 (1.05–3.88)*		2.56 (1.09–6.15)**
	150–600	1		--
	> 600	1.26 (0.71–2.23)		1.24 (0.58–2.65)
Albumin				
	< 4	1		
	$\ge$4	0.78 (0.46–1.27)		

S-HR: Sub-hazard ratio; HPD region: highest posterior density region; SD: Standard deviation; SGOT: serum glutamic-oxaloacetic transaminase; SGPT: serum glutamic-pyruvic transaminase; FBS: fasting blood sugar; ALP: Alkaline Phosphatase; iPTH: intact parathyroid hormone

* Significant at α = 0.2, ** Significant at α = 0.1

As indicated in Table 3, when the outcome was time to receive kidney transplantation, potassium level$>$5 mEq/L and phosphorus$\ge$5 mg/dL were statistically significant in the univariate analyses. In the Multivariable analysis, potassium level$>$5 mEq/L was associated with prolonging time to kidney transplantation. Holding the effect of all the other variables constant, the adjusted S-HR for potassium level$>$5 mEq/L was 0.28 (90% HPD region: 0.10–0.86). It is noteworthy that PH assumption was satisfied in data (results not shown).

**Table 3:** Univariate and Multivariable Competing Risks Model for Transplantation in ESRD Patients (n = 109)

Variables		Univariate		Multivariable
		S-HR (80% HPD Region)	Adjusted S-HR (90% HPD Region)	
Age		1 (0.97–1.04)	1 (0.96–1.03)	
Ferritin		0.999 (0.99–1.001)		
Creatinine		0.98 (0.95–1.04)		
Cholesterol		0.998 (0.99–1.01)		
Uric Acid		1.18 (0.88–1.58)		
SGOT		0.98 (0.92–1.05)		
SGPT		0.98 (0.94–1.003)		
‌Sodium		0.99 (0.98–1.02)		
‌FBS		1.01 (0.99–1.02)		
Sex	Male	1.94 (0.53–6.89)	2.81 (0.65–11.45)	
Hemoglobin				
	$\le$10	0.98 (0.44–2.24)		
	10-12.5 > 12.5	1 0.51 (0.19–1.29)		
Potassium				
	3.5-5	1	--	
	> 5	0.22 (0.1–0.45)*	0.28 (0.1–0.86)**	
ALP				
	$\le$300	1		
	> 300	0.8 (0.4–1.64)		
Calcium				
	$\le$8.6	2.41 (0.98–7.33)		
	8.6–9.5 $\ge$9.5	1 1.03 (0.32–3.6)		
Phosphorus				
	$<$5	1	--	
	$\ge$5	0.33 (0.16–0.72)*	0.59 (0.19–1.71)	
iPTH				
	$\le$150	0.32 (0.13–0.86)		
	150–600	1		
	> 600	0.57 (0.25–1.5)		
Albumin				
	$<$4	1		
	$\ge$4	0.86 (0.25–2.38)		

S-HR: Sub-hazard ratio; HPD region: highest posterior density region; SD: Standard deviation; SGOT: serum glutamic-oxaloacetic transaminase; SGPT: serum glutamic-pyruvic transaminase; FBS: fasting blood sugar; ALP: Alkaline Phosphatase; iPTH: intact parathyroid hormone

* Significant at α = 0.2, ** Significant at α = 0.1

## Discussion

It is critical to utilize an appropriate statistical method to detect factors that could potentially predict the prognosis of the disease [12]. In this study, the application of the competing risks analysis revealed that variables such as sex, serum calcium level $\ge$9.5 mg/dL, and iPTH level $\le$150 pg/mL had a significant effect on time to death.

The 4-year cumulative incidence of death in HD patients was estimated at 63.7%, while this estimation is reported to be about 75% in the most extensive multicenter study in Khuzestan province, Iran [5]. The estimated CIF in our analysis was roughly comparable to US registries but substantially lower than the Khuzestan province report, though it was still higher than European and Japanese registries [13, 14]. In another single-center study conducted by Ossareh et al. on 540 HD patients in Tehran, cumulative death incidence at 5 years was 53.7%, and survival was better in patients who started HD later (after 2004) [15]. Thus, the better survival of our patients compared to the Khuzestan province report in 2012 may be explained by improved HD technologies or advancements in pharmacological therapies in recent years. However, differences in genetic, economic, and demographic factors may also be involved.

Our results demonstrated that the male gender was associated with a higher risk of mortality. Similarly, others concluded that despite females having a greater prevalence of CKD, male HD patients had a higher mortality rate [12]. Cardiovascular events are one of the most important causes of death in HD patients. Therefore, the increased mortality rate in male HD patients may be explained by the higher rate of cardiovascular problems in the male population. Some studies have reported a higher rate of kidney transplantation in male HD patients than in females [13].

Age-associated factors like cardiovascular problems, malnutrition, or life-threatening comorbidities decrease the survival rate of older adults on HD. Some studies demonstrated a higher risk of death in old-aged HD patients [16, 17], while others found no significant association between age and higher mortality risk [18, 19]. Our findings showed a slight increase in the hazard of death for every year of age increase, although it was not statistically significant in the multivariable model.

In many former studies, hypocalcemia and hypercalcemia were both associated with a higher risk of disease progression and mortality in HD patients [20–23]. Similarly, our results indicated that the adjusted hazard of death increases in patients with serum calcium levels $\le$8.6 mg/dL and serum calcium level $\ge$9.5 mg/dL with respect to the reference group (8.6–9.5 mg/dL). However, only the effect of serum calcium level $\ge$9.5 mg/dL was statistically significant. The association between high calcium levels and high mortality may be due to the interaction of calcium with other factors like hyperphosphatemia. In 2016, in a large multicenter study in 20 provinces of Iran, it was reported that hypercalcemia and hyperphosphatemia were observed in 21% and 34% of HD patients, respectively, and only 8% of patients reached all target ranges recommended by the National Kidney Foundation Dialysis Outcomes Quality Initiative (K/DOQI) guidelines [24]. Development of cardiovascular disease is accelerated by an increased risk of vascular calcification caused by elevated extracellular calcium levels and hyperphosphatemia [25]. Besides cardiovascular events, Nakano et al. reported that hypercalcemia is related to a higher risk of infection-related death [26].

Controlling the phosphorus levels during HD is the most critical and challenging clinical target because untreated hyperphosphatemia can lead to bone pain, pruritus, worsening secondary hyperparathyroidism, and an increased risk of cardiovascular-related mortality [27, 28]. In this study, Phosphorus levels higher than 5 mg/dL were associated with an elevated hazard of death; however, it was not statistically significant. Ossareh et al., in a study in Iran, reported that there was only a significant effect of hyperphosphatemia on the survival of HD patients when a time-dependent model was used [15]. Further investigations with a larger sample size and repeated measurements of laboratory parameters during HD are required for reaching a precise conclusion about phosphorus.

Abnormal metabolism of parathyroid hormone is one of the major CKD-MBD complications. A U-shape PTH-mortality relationship has been recognized in HD patients with increased mortality with both higher and lower than expected values [25, 29]. Interestingly, we did not find an elevated mortality rate associated with high iPTH levels. However, our results demonstrated that the adjusted sub-hazard of death for HD patients with iPTH levels $\le$150 pg/mL was higher than those with iPTH levels between 150 and 600 pg/mL. Due to high bone turnover in HD patients, iPTH levels should be managed therapeutically to ensure they do not fall below normal values. Several studies in patients with advanced CKD revealed associations between low iPTH, regarded as a surrogate indicator of adynamic bone disease (ABD), and arterial calcification, with significant interactions with pathogenetic factors, such as calcium load, inflammation, or malnutrition [30]. Published studies showed that all of the conditions mentioned above are directly or indirectly connected to higher mortality [1, 31]. In a study on 7191 HD patients in 58 HD centers in Iran, it was reported that 46% of patients had iPTH levels of $\le$150 pg/mL, and ABD is a more significant problem than hyperparathyroidism (observed in 19% of the population) [24].

It was reported that high albumin levels were protective factors, reducing the hazard of death in HD patients [1]. This finding could be likely explained by considering higher albumin levels as an indicator of good nutrition. According to recent researches, serum albumin rather represents a condition of inflammation and has limited value as a marker of nutrition status [32]. Previous researches illustrated that the complex malnutrition-inflammation is associated with low levels of iPTH in HD patients [33]. In the current study, serum albumin had no significant effect on time to death incidence. This might be due to concurrent low levels of iPTH or unobserved C-reactive protein. Saeedi et al. also reported no significant impact of high albumin levels on HD patients’ survival in a study utilizing a complex competing risks model [12].

The hazard of going under kidney transplantation had a decreasing trend (Fig. 2), showing that transplantation was more frequent in the early years of HD initiation. In the first 2 years, as shown in Fig. 3[C], the cumulative incidence of undergoing kidney transplantation was greater than death incidence. Many ESRD patients prefer transplantation as soon as possible since studies have demonstrated that the shorter the time spent on dialysis, the better the patient and graft survival rates. Iran has one of the most successful kidney transplantation programs, and in recent years, approximately 2500–2700 kidney transplants performed per year [34]. Hence, patients who do not have contraindications to transplantation can be transplanted in a relatively short time.

Serum potassium might affect graft failure after kidney transplantation, and post-transplant hyperkalemia may result in serious complications such as ICU hospitalizations [35]. Our analysis indicated that potassium level > 5 mEq/L lessens the time to kidney transplantation. Clinically, it does not appear that such a relationship exists, and it has not been investigated in other studies.

Our study limitations could be a small sample size, lack of adjustments for serum vitamin D level, fibroblast growth factor 23, time of diagnosis of the disease, and perhaps residual confounders. Moreover, we did not evaluate the survival of patients based on their underlying disease (e.g., diabetes mellitus), residual renal function, and dialysis adequacy. Taking into account both death and transplantation simultaneously could be a study’s strengths, as well as the correlation between the events.

## Conclusion

In this single-center study, male gender was a non-modifiable risk factor for mortality. Moreover, high serum calcium, and low iPTH levels were associated with worse outcomes. Correcting these laboratory parameters may improve patients’ survival in the HD population. While hypercalcemia and hypoparathyroidism were linked to an increased hazard of death, we found no significant effect of serum phosphorus or serum albumin levels in patients with ESRD. In addition, the flexibility of the improper form of the two-parameter Weibull model for evaluating significant laboratory factors was demonstrated. This method was more versatile than the traditional methods since it considers the event of interest and the competing event, as well as the correlation between them simultaneously.

## Data Availability

The datasets used for this study are available from the corresponding author upon request.

## Abbreviations

CKD: Chronic kidney Disease
HD: Hemodialysis
ESRD: End-Stage Renal Disease
LMICs: Low- and middle-income countries
CKD-MBD: Chronic kidney disease-mineral and bone disorders
iPTH: Intact parathyroid hormone
eGFR: Estimated glomerular filtration rate
PH: Proportional hazards
SGOT: Serum glutamic-oxaloacetic transaminase
SGOT: Serum glutamic-pyruvic transaminase
FBS: Fasting blood sugar
ALP: Alkaline phosphatase
SD: Standard deviation
MCMC: Markov Chain Monte Carlo
HPD: Highest posterior density
CIF: Cumulative incidence function
S-HR: Sub-hazard ratio.
